# Biofilms as protective cocoons against biocides: from bacterial adaptation to One Health issues

**DOI:** 10.1099/mic.0.001340

**Published:** 2023-06-02

**Authors:** Raphaël Charron, Marine Boulanger, Romain Briandet, Arnaud Bridier

**Affiliations:** ^1^​ Antibiotics, Biocides, Residues and Resistance Unit, Fougères Laboratory, French Agency for Food, Environmental and Occupational Health & Safety (ANSES), 35300 Fougères, France; ^2^​ Université Paris-Saclay, INRAE, AgroParisTech, Micalis Institute, 78350, Jouy-en-Josas, France

**Keywords:** antimicrobial resistance, bacterial adaptation, biocides, biofilm, food chain, One Health

## Abstract

Bacteria in the food chain mostly live in communities associated with surfaces known as biofilms, which confer specific survival and adaptive abilities. In such communities, the bacteria mostly exhibit higher tolerance to external stress, and their recurrent exposure along the food chain to biocides used during cleaning and disinfection procedures raises concern about the adaptation routes they develop, both at single-cell and communal levels. In recent years, an increasing number of research subjects have focused on understanding the specific features of biofilms that enable bacterial populations to adapt to biocide exposure within a ‘protective cocoon’. The first part of this review concentrates on the diversity of adaptive strategies, including structural modulation of these biofilms, physiological response or the acquisition of genetic resistance. The second part discusses the possible side effects of biofilm adaptation to biocides on antimicrobial cross-resistance, virulence and colonization features from a One Health perspective.

## Introduction

The increase in antimicrobial resistance over the past 60 years has become a major public health concern and has raised questions about the drivers of this emergence of resistant bacteria. Many review papers have focused on antibiotics used to treat bacterial infections and the link between antibiotic use, resistance selection and underlying mechanisms in many areas [1–3]. However, other substances — such as biocides — are used daily to control bacterial contamination of surfaces in diverse environments and to prevent the transmission of pathogens to humans or animals. Unlike antibiotics, which often have a specific mode of action based on one or only few bacterial targets, chemical biocides mostly act on multiple targets [4]. Through a variety of complex mechanisms, resistance to most of the biocides in use has already been documented [5]. Because of the large-scale and repeated use of biocides, resistant bacteria can quickly propagate and colonize new environments, thereby accelerating the spread of resistant clones. In 2014, Zou *et al*. documented the prevalence of several quaternary ammonium compound (QAC) resistance genes in *Escherichia coli* isolated from retail meat and identified the major prevalence of five genes, reaching 100 % for *ydgE/F* genes [6]. However, it is important to clarify that the term biocide resistance is still confusing as it encompasses various definitions, illustrating a high diversity of practical situations in terms of levels of susceptibility loss [5]. If the term antibiotic resistance is usually based on the clinical concentration used, biocides are in general used at high concentrations where the vast majority of bacteria are eliminated. Some resistances are in fact just describing a decrease of susceptibility for the bacteria, which would not survive efficient treatment, at an in-use concentration. A consensus in the scientific community to define which threshold could be used to differentiate between a susceptible and a resistant strain is still required. However, susceptibility reductions could still confer an advantage to the cells with inaccurate application of the treatment, which would ultimately increase the risks of human infections and should thus be considered as seriously as the evolution of antibiotic resistances. In this context, resistance to biocides also needs to be taken seriously because of the mechanisms that potentially confer cross-resistance among the two families of molecules, i.e. biocides and antibiotics. Indeed, although both this phenomenon and its importance in the development of antimicrobial resistance need to be fully understood, several studies have demonstrated a relationship between biocide exposure and antibiotic resistance selection [7–9]. For example, multidrug efflux systems [10–14], cell wall permeability modifications [12, 13] or alterations in metabolic cascades [15] have all been associated with bacterial resistance to both antibiotics and biocides. It is thus of prime importance to understand the evolution and adaptation of bacteria to biocide actions so as to unravel possible side effects on global resistance to antimicrobials (including antibiotics). Studies on other possible impacts of biocides on dissemination of antibacterial resistance, colonization of new environments and bacterial virulence also need to be taken into account when identifying the One Health effects of the use of biocides.

In their natural environments, most microorganisms live in spatially organized communities called biofilms, which are associated with surfaces [16]. In these biostructures, microbial cells are embedded in self-produced extracellular polymeric substances (EPSs). This matrix is typically composed of water and a mixture of polysaccharides, DNA, proteins and lipids [17]. This organic slime confers broad protection to the bacteria against environmental stresses such as dehydration or exposure to antimicrobial compounds [18–20]. Molecular diffusion/reaction limitations participate in biofilm resistance because gradients of nutrients and oxygen are induced in the 3D structure. As a consequence, biofilm phenotypes tend to be highly heterogeneous compared with planktonic populations, since different bacterial subpopulations adapt to their local microenvironments [21]. While bacteria living in the external layers grow at a normal speed, those in the inner layers are often subject to the activation of stress responses triggered by local nutrient depletion, leading to the appearance of particular subpopulations such as persisters or viable but non-culturable (VBNC) cells [22]. This heterogeneity can also influence bacterial survival, as some of these populations become highly tolerant or even resistant to curative treatments [23].

The recurrent exposure of biofilms to biocides in multiple environments has led us to explore in this review the adaptation routes of bacteria at cellular and populational scales to understand how biofilms can drive resistance. The second part of this paper explores, from a One Health viewpoint, the possible side effects that adaptation to biocides could have in newly resistant subpopulations in terms of antimicrobial cross-resistance, virulence and colonization features.

## Structural modulations of biofilms in response to biocides

**Fig. 1.**
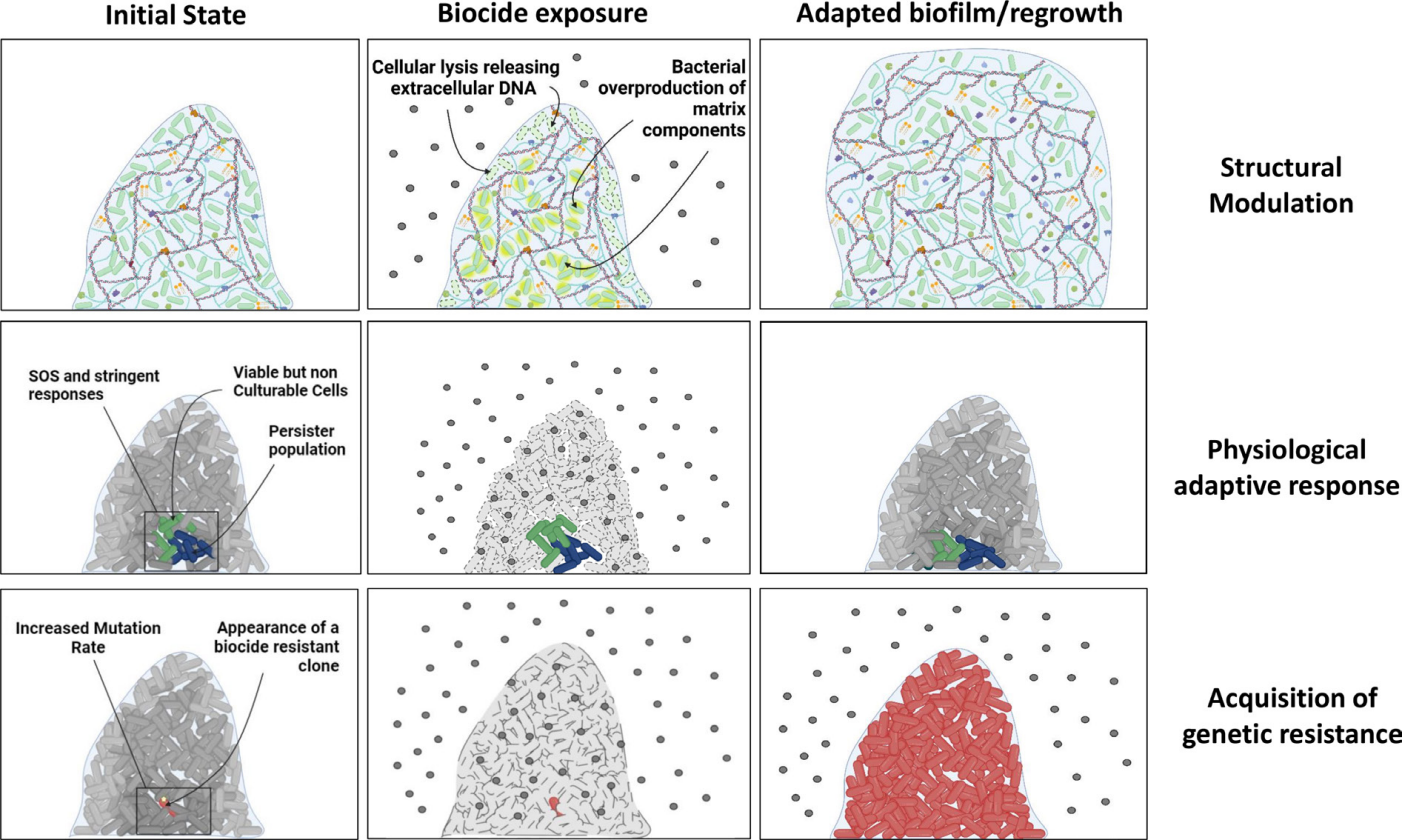
Structural modulation: exposure to biocides induces stress responses, which lead to upregulation of matrix component production. Cells lysed by the biocide release their eDNA into the biofilm, thus reinforcing it. Physiological adaptive response: nutrients and oxygen gradients lead to specific physiological states, such as persister cells or VBNC cells. These cells can tolerate temporary exposure to biocides, while the rest of the biofilm will be eliminated. If the exposure to biocides stops, the surviving cells will again become active and regrow a new biofilm, with new persister populations in the inner layers. Acquisition of genetic resistance: stressful conditions in the inner layers can increase the mutation rate, which can randomly lead to the appearance of a biocide-resistant clone. If this clone is then exposed to a biocide, all the other cells will be eliminated while this cell will survive and develop a new biofilm, independently of the presence or absence of the biocide.

Biofilms are dynamic structures whose shapes and sizes are driven by the bacterial responses to environmental conditions and stresses. Bacteria can adapt to changing conditions through regulation of matrix component production, biofilm spatial organization, cannibalism or vascularization [24]. In this part, we will focus on the biofilm’s structural plasticity as a response to biocide exposure, especially through the modulation of extracellular matrix composition and the bacteria’s collective behaviour (Fig. 1) .

### Intrinsic features of the extracellular matrix

The structural integrity of a biofilm is maintained through an extracellular matrix (ECM) produced by bacteria during biofilm maturation. Its composition can differ regarding the species embedded in the biofilm, local stresses or nutrient availability. It can be composed of water, polysaccharides, proteins, lipids, surfactants, glycolipids, extracellular DNA (eDNA), membrane vesicles and ions, all in various quantities. However, polysaccharides, proteins and eDNA are the three main components found for many bacteria [17, 25]. If bacteria are considered bricks, then ECM is the cement that provides mechanical biofilm resistance and can mitigate biocide effects within the 3D structure [17, 26].

Spatiotemporal patterns of inactivation in the biofilm architecture are highly biocide- and strain-specific, as demonstrated by time-lapse confocal laser scanning microscopy approaches [27–29]. Penetration of the 3D biofilm structure by biocides depends on interactions between the biocide molecules and ECM components, and can be slowed through the sorption of positive/negative charges or hydrophobic interactions. For instance, it takes about 25 min for glutaraldehyde to diffuse inside a *Staphylococcus epidermidis* biofilm, whereas its antimicrobial action in the absence of sorption reactions is estimated to be 24 s [27]. Sometimes, antimicrobial substances cannot even penetrate the biofilm to reach their biotargets, bacteria. For example, a synergistic combination of the biochemical nature of the ECM and biofilm roughness and topography has been shown to confer non-wetting properties to *Bacillus subtilis* biofilm surfaces [30]. It can be viewed as an impermeable coat that prevents antimicrobial actions, as biocides are not able to fully penetrate the biofilm [30]. Kobayashi’s team indicated that the amphiphilic protein BslA could be partially responsible for the surface repellency of *B. subtilis* biofilms by forming a protective monolayer at the biofilm’s interface with air [26].

Upon exposure to a biocide, the structural organization of a biofilm can be modified directly as a consequence of biocide action or through the defensive adaptive responses of bacteria. The main mechanisms involved in such modulations are described below.

### Modulations of extracellular matrix composition

As mentioned above, the ECM is composed of different macro- and micro-molecules according to various conditions, including which strains are embedded in the biofilm and environmental fluctuations. In response to biocide exposure, it is also common to observe an increase in biofilm production, characterized by an increase in the ECM that acts as a protective shield for bacteria, which are thus exposed to sublethal biocide concentrations, allowing their survival. Modulation of the biofilm density has already been associated with increased survival against different biocide families [31]. After being stressed by sublethal doses of ClO_2_, the quantity of matrix has been shown to increase in biofilm formed by *B. subtilis* [32]. As another example, the crystal violet assay, commonly used to graduate biofilm formation, has shown that uropathogenic *E. coli* displayed a significant increase in biofilm production, especially after exposure to triclosan and benzalkonium chloride (BAC) [33]. It has also been shown that a previous adaptation by *E. coli* to biocides sodium nitrite and sodium hypochlorite could enhance biofilm formation with or without further exposure to a biocide [34]. The production of EPSs such as polysaccharides is one of the most frequent responses to biocide exposure. A large number of biocides have been shown to induce EPS production in different bacterial species through the activation of different pathways and regulators [32, 35, 36]. Polysaccharides are not the only component activitated during biocide exposure, as the overproduction of amyloid fibres has also been associated with the exposure of biofilms to different classes of biocides, e.g. the production of Curli proteins in *E. coli* biofilm exposed to various biocide substances, including glutaraldehyde, isopropanol, chlorophene and chlorhexidine [35].

### Role of extracellular DNA in interfering with biocide action

eDNA is another major component of the ECM released through cell lysis, membrane vesicles or dedicated secretion systems [37]. It can remain in the environment for some time until degradation or effective removal by mechanical and chemical action. It is of real importance for bacteria, as it can be a source of resistance genes through horizontal gene transfer as described further below. Its involvement in biofilm adhesion to surfaces and its ability to interact with the bacterial cell wall has already been demonstrated by its anionic properties that allow binding to positively charged molecules, proteins or polysaccharides [38]. Jennings *et al*. showed that in the *Pseudomonas aeruginosa* matrix, the presence of partially acetylated GalNAc and GlcNAc on a cationic exopolysaccharide Pel allows cross-links with eDNA, which is therefore partially responsible for the integrity of the biofilm’s structure [39]. This negative charge could also be involved in the capture of cationic biocides, which could limit penetration through to the deepest biofilm layers and decrease antimicrobial efficiency. Such a process has already been observed with cationic antibiotics [40, 41] and antimicrobial peptides [25], and is likely to occur with positively charged biocides such as QACs. Moreover, the amount of eDNA produced, which is directly linked to its release in the biofilm ECM, can be promoted with the use of biocides. In *P. aeruginosa* and *Staphylococcus aureus *, Moshynets *et al*. [42] showed an increase in eDNA release after exposure to alcohols, hydrogen peroxide (H_2_O_2_) and QACs resulting from exposed cell lysis.

### Production of protective exoenzymes

Inactivation by enzymes has not yet been fully described in the literature but exoenzymes synthesized and released into the ECM may be another strategy used by biofilms to combat oxidative biocide exposure (e.g. hypochlorite solutions). Oxidoreductase enzymes such as catalase and superoxide dismutase can be expressed and released into the exopolymeric matrix by certain bacterial species entrapped in the biofilm. They can be exploited by enzyme non-producing bacteria, thereby behaving as public goods [43]. Their presence in the ECM can be stimulated after exposure to oxidative biocides. Exposure to 5 mM sodium hypochlorite (NaOCl) or 50 µM H_2_O_2_ led to a higher catalase activity in *S. epidermidis* biofilm compared with their planktonic homologues [44]. It has also been demonstrated that penetration of *P. aeruginosa* catalase-deficient (*katA* gene knock-out) biofilms by the active form of H_2_O_2_ was facilitated when compared with the wild-type isogenic strain, suggesting that catalase activity plays a protective role [45]. Disruption of the *katA* gene has also been shown to affect *Proteus mirabilis* biofilm sensitivity to H_2_O_2_ through the modification of matrix composition, such as a reduction in carbohydrate content [46]. Similarly, superoxide dismutases have been shown to protect bacteria from the action of H_2_O_2_. In *E. coli *, the *sodC* chromosomal gene encoding superoxide dismutase confers higher survival rates against H_2_O_2_, while also being associated with increased biofilm formation [47].

### Quorum sensing as a cooperative communication system

Bacterial cooperation plays a key role in biofilm adaptation to biocide exposure. This cooperation can be triggered by inducing quorum-sensing (QS) mechanisms present in many bacterial species [48]. This system is based on the secretion of signalling molecules by bacteria which will in turn interact with a receptor protein in neighbouring bacterial cells, regulating gene expression in the population above a critical threshold [49]. QS systems have been widely associated with every step of biofilm formation, from initial attachment to dispersal [50]. A deficient QS system has already been associated with a large defect in biofilm formation, as demonstrated by Sakuragi *et al*. [51] in *P. aeruginosa* biofilms, where mutants of *lasI* and *rhlI*, which are QS effectors, were severely hampered in their ability to produce exopolysaccharide components. QS systems are essential for coordinating a cooperative mutualistic action. Synergistic biofilm formation between two different species also require an efficient QS system, as described by Rickard *et al*. [52], where the AI-2 molecule was required by *Actinomyces naeslundii* and *Streptococcus oralis* to build a common biofilm structure. Additionally, in Gram-positive cells, the release of eDNA inside the bacterial structure can also be mediated by a QS molecule called the CSP (competence-stimulating peptide), which regulates the expression of an autolysin, LytA, and two putative bacteriocins, triggering cell lysis and consequently DNA release. The CSP is essential for stabilizing the biofilm structure in several species of the genus *Streptococcus* [53]. By regulating the biofilm structure and components, QS can therefore enhance the chances of bacteria surviving exposure to a biocide, fostering bacterial cohesion in order to form a structured, protective matrix. The use of furanone, an inhibitor of the QS molecule AI-2, has already been associated with increased efficiency of hypochlorite and BAC against *Salmonella enterica* [54]. QS molecules LasR and RhlR have also been associated with a decreased susceptibility of *P. aeruginosa* against oxidative stressors such as H_2_O_2_, with the regulation of catalase and superoxide dismutase genes [55]. Finally, direct responses to biocide stress through QS regulators have also been reported. In fact, Gholamrezazadeh *et al*. reported that the use of BAC, nanosilver and acid-based formulations induced the expression of the QS regulator RhlR in *P. aeruginosa* isolates [56].

### Role of ecological interactions

In their natural environments, mono-species biofilms can be found in specific biotopes, but the vast majority of biofilms are multi-species [57]. Microbial species are not spatially distributed randomly in a diversified biofilm. Beneficial or negative interactions between species can lead to a specific arrangement of the biofilm surface area, and can depend on morphological or bacterial structural properties [58]. The spatial organization has an impact on the biofilm’s structural ability to resist a stress such as exposure to a biocide [59]. Microorganisms can also use specific species-related interactions as an adaptive strategy to counter biocide exposure. Some species can be protected by others from exposure to and the action of biocides within the biofilm’s 3D structure [29, 60, 61]. As mentioned above, it is quite common to observe an increase in ECM production in single-strain biofilms exposed to biocides. The same is true when biofilms comprise multiple strains. In a dual-species biofilm, the production of EPSs can be increased, thus acting as a more efficient protective shield. Pang *et al*. demonstrated that more EPSs were produced in a biofilm composed of *P. aeruginosa* with non-cellulose-producing *Salmonella enterica* serovar Enteritidis [62], while Burmølle *et al*. demonstrated a synergistic interaction between four different species in response to hydrogen peroxide [63]. Some pathogenic strains of *E. coli* have already been shown to be able to increase the production of EPS components by other non-pathogenic strains via the secretion of matrix-stimulating metabolites [64]. Based on the hypothesis that the biochemical composition of the ECM is even more heterogeneous in a multi-species biofilm, this may increase biocide deactivation by diffusion and/or sorption reactions [61].

The complexity of a multispecies biofilm’s reactions to biocide exposure also resides in its production of biomolecules. The natural diversity of microorganisms in biofilms reflects a full range of biomolecules found in the ECM, including cooperative public goods [65]. This phenomenon may allow susceptible bacteria that do not produce biomolecules to become tolerant to antimicrobials simply by the presence in its environment of bioproducts synthesized by its neighbouring bacteria. For example, *P. aeruginosa* is able to produce SdsA1, an SDS-hydrolase enzyme [66] which, produced individually by a single strain, can protect the whole community in a mixed-species biofilm [59]. Similarly, the matrix component produced by *B. subtilis* NDmed protects *S. aureus* from the actions of disinfectants [60]. In the event of environmental stresses such as starvation or antimicrobial addition, some subpopulations inside biofilms can even enter a specific lifestyle, including cannibalism. In *B. subtilis *, some subpopulations inside biofilms have been shown to secrete toxins able to lyse neighbouring non-immunized cells, which would then release nutrients inside the biofilm. Interestingly, the cells that could enter this cannibal state are the same ones that previously produced the ECM [67]. It has also been shown that selected bacilli swimmers can infiltrate and vascularize *S. aureus* biofilm by creating transient pores, offering direct routes for antimicrobials to reach the deepest populations of biofilm communities [68, 69]. In conclusion, intra- and inter-species interactions play an important, but still largely unexplored, role in modulating the spatial organization of the biofilm’s structure and functions.

## Bacterial physiological modulations and active responses

One of the main differences between biofilms and planktonic cultures is their divergence in phenotypes. While planktonic cultures are usually exposed to homogeneous conditions and therefore have similar phenotypes, biofilms are composed of a multitude of microenvironments, exposing some of the cells in the inner layers of the biofilm to very low concentrations of nutrients and oxygen, consequently increasing the heterogeneity of physiological states [70]. These conditions promote stress responses in biofilms that completely remodel the physiology of these cells, triggering differential gene expression and thus new phenotypes, including reduced growth and potentially increased tolerance. Among these phenotypes, the development of persisters and VBNC cell populations is frequent in biofilms [22]. These populations are marked by their inability to grow, and are often challenging to eradicate due to the inactivation of certain antimicrobial targets. These recalcitrance phenomena often occur during antibiotic treatments because of the target-specific nature of antibiotics. For biocides, the multiplicity of targets usually prevents these limitations on planktonic cells. However, their diffusion-reaction limitation in biofilms can reduce the number of impacted targets for some treatments [71]. A recent study by Fernandes *et al*., described the ability of *Pseudomonas fluorescens* and *Bacillus cereus* biofilm populations to regrow after different biocide treatments [72]. They observed that persister populations were able to survive BAC, glyoxal and glycolic acid treatments. *B. cereus* endospores were quite resilient to biocide treatments, while a considerable number of VBNCs were found among the surviving *P. fluorescens* populations. Peracetic acid (PAA) had a stronger effect, being able to eliminate all the *B. cereus* population, but not *P. fluorescens* VBNCs. The surviving populations were able to regrow in planktonic and biofilm conditions but the new population usually had the same susceptibility as the initial one, demonstrating that their survival was only due to their transitory persisting metabolism. Some studies have demonstrated that exposure to biocides can trigger the emergence of a VBNC population [73, 74]. In this section, we will focus on the different stress responses and physiological modulations in the biofilm that can lead to biocide-resilient populations (Fig. 1).

### The stringent response

The stringent response is a key regulatory mechanism within bacteria. This stress response is activated in the event of nutrient depletion [75] to remodulate the cellular functions and limit gene expression to those that are essential. Thus, the inner layers of biofilms can be subject to a stringent response triggered by the lack of nutrients in certain strata or niches. Briefly, the stringent response can be induced through two homologous proteins: RelA and SpoT. These two proteins are activated through different external stresses, such as carbon and lipid starvation or internal consequences, such as amino acid deprivation [18]. They are both able to synthesize an alarmone called (p)ppGpp. This molecule targets many cellular functions, being involved in nucleotides, lipids, phosphates and amino acid metabolism, and acting on DNA replication, DNA transcription and RNA translation [76]. Through this process, bacteria can reduce their activities and focus on essential functions such as amino acid biosynthesis and nutrient uptake [75], while inhibiting growth processes such as replication, transcription and translation [77]. Thus, the stringent response often occurs in the inner layers of biofilms where nutrient availability can be reduced. Many studies have shown that the stringent response has, in addition, a direct regulatory role in biofilm formation and dispersion, being involved in the regulation of motility and EPS production [78, 79]. Within biofilms, the stringent response has been associated with an increased bacterial tolerance to antibiotics [80]. Similar effects are observed with biocides, and results from the inhibition of their targets are in keeping with the inhibition of cell functions. Both reduced growth rates and nutrient limitation have been associated with reduced sensitivity to biocides, demonstrating a possible effect of the stringent response on biocide tolerance [81–83]. The stringent response has also been shown to control the activity of catalases and hence to mediate the survival of bacteria against oxidative stressors, as demonstrated with hydrogen peroxide [84, 85]. A link between chlorhexidine and the stringent response has also been observed in *Enterococcus faecium* [86]. Recently, another study led by Nordholt *et al*. [87] used BAC to periodically disinfect *E. coli *, the goal being to simulate industrial conditions where bacteria are routinely exposed to biocides. They indicated that this periodic process induced a bacterial tolerance strongly linked to the triggering of the stringent response.

### SOS response

The SOS response is a bacterial defence system involved in the restoration of damaged DNA. It is controlled by more than 50 genes regulated by inducer RecA and repressor LexA. Activated when ssDNA is starting to accumulate in the cell due to a polymerase being unable to fulfil its function while a helicase continues to unbind DNA upstream, RecA will induce an autocleavation of LexA and derepress the genes involved in the SOS response [88]. These genes code for polymerases that will repair the damaged DNA and therefore provide a level of tolerance to the deleterious effects of some biocides. The general environmental stress to which bacteria evolving in biofilms are exposed promotes the SOS response. In fact, six genes of the SOS response have already been positively associated with the accumulation of (p)ppGpp in *E. coli *, including RecA [89], demonstrating a link between stringent and SOS responses. The constant activation of the SOS response could hence be another argument for affirming that biofilms are a favourable condition for the emergence of tolerant clones. Several biocide families target DNA, and their effects may thus be directly impacted by the SOS repair system. In fact, the use of polyhexamethylene biguanide (PHMB) has already been associated with the triggering of DNA repair systems [90]. Peroxide-based biocides, such as PAA and H_2_O_2_, induce the appearance of reactive oxygen species (ROS), which damage DNA. These two biocides have already been associated with the triggering of an SOS response [91, 92]. In a similar manner, chlorine-releasing compounds such as NaOCl and chlorhexidine can also increase ROS production and have also been associated with instigation of the SOS response [13, 92, 93].

### Toxin–antitoxin modules

Toxin–antitoxin modules are also systems able to promote the emergence of bacterial tolerance. These modules work through the action of a toxin, which inhibits several essential cellular processes such as DNA synthesis, DNA translation and cell wall synthesis [94]. The effects of these toxins are regulated by the antitoxins binding them, which are degraded in the event of external stress [18]. Thus, the toxin can be released and interfere with cellular functions, which will in turn slow bacterial growth and reduce biocide targets. Toxin–antitoxin systems are usually closely linked to stringent and SOS responses. In fact, the TisB/IstR-1 system is regulated by RecA and thereby strongly associated with the SOS response. This system has been associated with persister formation by the regulation of the proton motive force leading to a reduction of ATP in the cell, which will shut down multiple targets [95]. The HipA/HipB toxin–antitoxin system has itself been associated with (p)ppgpp and the stringent response. Two hypotheses have been forwarded regarding the way this system functions. One has suggested that HipA phosphorylates elongation factor EF-Tu, which ultimately leads to growth inhibition [96], while the other has rejected this hypothesis and has instead suggested that HipA phosphorylates the glutamyl-tRNA-synthetase GltX [97]. This phosphorylation interferes with the aminoacylation of tRNA-Glu, which directly leads to the accumulation of uncharged structures in the ribosomal A site, triggering RelA and the subsequent synthesis of (p)ppgpp. Logically, biofilms are thus a privileged zone for the development of toxin–antitoxin responses and the formation of persister cells [98]. Furthermore, research tends to show that toxin–antitoxin systems can even promote biofilm formation [99–101]. While no articles have directly linked the tolerance of bacteria in biofilms to biocides through toxin–antitoxin systems, some nonetheless have shown a link between the presence of some of these systems and the emergence of biocide tolerance. The PemI/PemK system has been identified on a chlorhexidine-tolerant *Klebsiella pneumoniae* plasmid [102]. Another study, performed on *Acinetobacter baumannii *, showed an association between the AbkA/AbkB system and chlorhexidine tolerance [103].

### Active and passive trans-membrane transport

Efflux is an active pumping mechanism by which bacteria reject molecules that enter their inner structure from their environment, thus enabling the bacteria to survive. Efflux pumps are therefore strongly associated with tolerance, and have already been shown to be upregulated in dormant bacteria [104]. In addition, efflux pumps and biofilms are closely linked [105, 106]. This could be explained by the use of some broad-spectrum efflux pumps in functions other than the rejection of antimicrobials that could drive biofilm formation, for example the secretion of ECM components produced by bacteria [105]. Some efflux pumps are also involved in the secretion of QS compounds, which play a major role in the communication of bacteria inside biofilms [105]. Among the different efflux pumps, some have already been associated with both biofilm formation and biocide tolerance, demonstrating that biofilm cells could be less susceptible to biocides. The resistance–nodulation–cell division (RND) type of efflux pump is predominant. This superfamily is composed of many multidrug efflux pumps usually with a broad efflux spectrum. The AcrAB system may be the most well characterized. It has been shown to be involved in tolerance to several classes of biocides in different species, including *E. coli* [107] and *K. pneumoniae* [108]. AcrAB also appears to be essential for biofilm maintenance at a high level [109]. Amongst the other RND efflux pumps, the AdeABC efflux system of *A. baumannii* or the MexCD-oprJ system of *P. aeruginosa* have also been associated with both biofilm formation [110, 111] and antimicrobial tolerance [112–114]. Other efflux pump families are also involved in both biofilm formation and biocide tolerance, such as the major superfacilitator superfamily (MFS) and the small multidrug resistance (SMR) families. The MFS efflux pump NorA, present in *Staphylococcus aureus *, has been involved in tolerance to chlorhexidine, cetrimide and BAC [115, 116], while also contributing to biofilm formation [117]. In the SMR family, the *Listeria monocytogenes* EmrE efflux pump confers tolerance to QACs [118] while being essential in biofilm formation [119]. Additionally, the SMR SugE efflux pump has been shown to confer greater tolerance to QACs in *E. coli* biofilms than in planktonic cultures [120].

Joint regulation mechanisms between passive and active transport also occur in bacterial cells. While efflux pumps are upregulated, porins —transmembrane channels used for internalizing molecules and nutrients — are downregulated to limit the entry of new antimicrobials and facilitate the externalization of those already inside [121]. For example, in *E. coli *, three global regulators – MarA, SoxS and Rob – regulate both porins and efflux systems at the same time, upregulating AcrAB, TolC and MicF, the last being a known repressor of OmpF [122]. Porins are usually essential for biofilm formation [123, 124], which is in opposition to resistance mechanisms, as upregulation facilitates the entry of antimicrobials into the cell. Indeed, porins can contribute to cell adhesion and autoaggregation [125, 126] but also to the export of matrix components, such as the Pel polysaccharide in *P. aeruginosa* [127]. This therefore contrasts with biofilm tolerance and biocide tolerance, as bacteria usually decrease porin expression during biocide exposure [128, 129].

## Acquisition of genetically mediated resistance

**Fig. 2.**
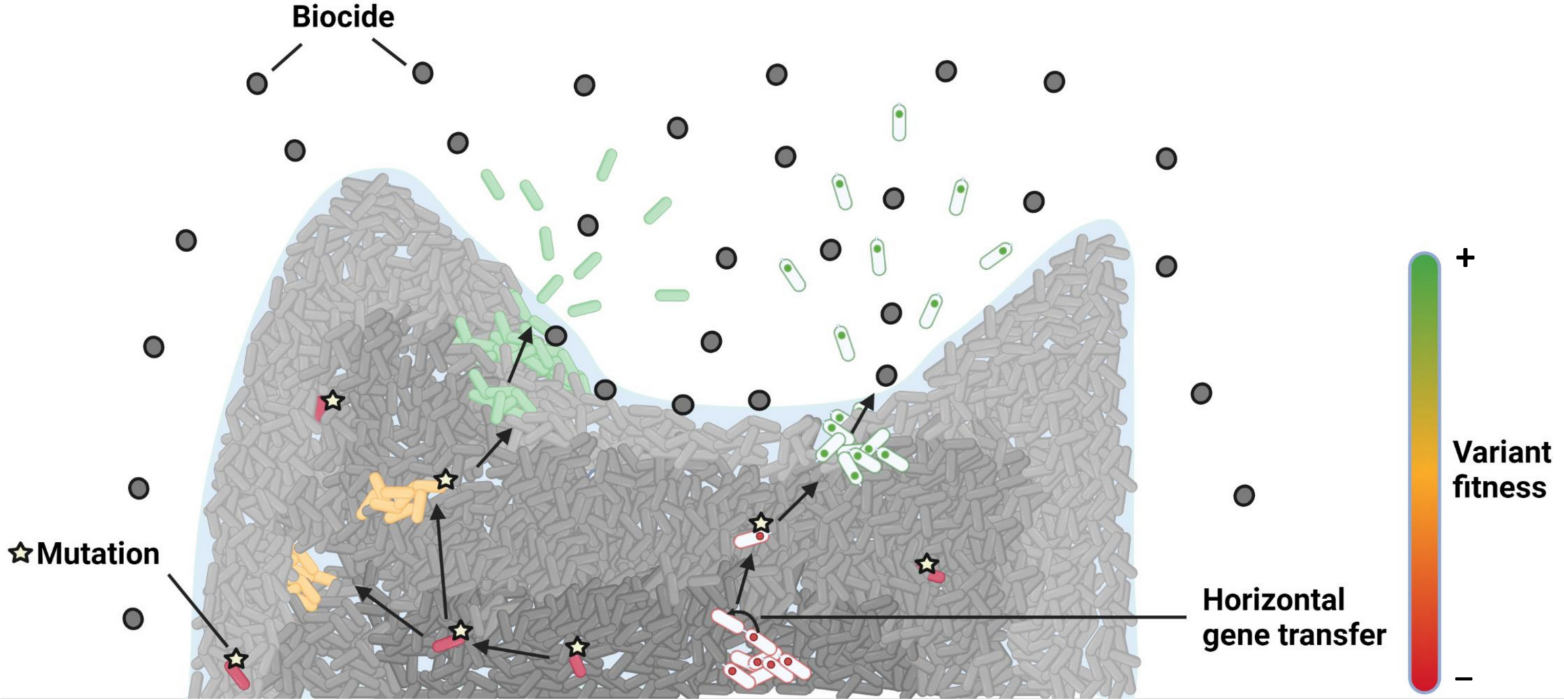
Resistance can occur through mutational events or plasmid carriage. However, it is often associated with a fitness cost. The biofilm protects unfit variants, which can become fit to multiply and finally disseminate in a biocide-full environment through compensatory mutational events.

Although modulation of the biofilm’s structure and the inner physiological state of certain populations inside the matrix can confer tolerance against biocides, they cannot be considered as resistance mechanisms, as the bacteria’s survival is only mediated by genetic regulation cascades which will disappear immediately once the stress is removed. In contrast, genetically mediated resistances are anchored in the genome and will last even if the bacteria disseminate in another less stressful environment. Genetically mediated resistances will also be transmitted to the bacterial daughter cells, spreading the resistance rapidly in the biotope. In this section, we will focus on how biofilms and biocide exposure can influence and select the emergence of biocide-resistant genetic variants, and how such resistant variants can fix and evolve in the protective environment conferred by the biofilm (Fig. 2) .

### From physiological adaptation to genetic resistance

In recent years, several studies have revealed a relationship between tolerance and the emergence of resistance. Indeed, some studies have shown that tolerance often precedes resistance in a bacterial cell population, especially when cells are exposed to high concentrations of antimicrobials [87, 130, 131]. The explanation for this phenomenon could be that the tolerant metabolism facilitates emergence of the resistant phenotype. In a bacterial population, a resistant phenotype often requires the previous appearance of several partial resistances that provide weaker protections but also entail fewer fitness costs. These are sometimes not strong enough to survive the antimicrobial attack, and this is where tolerance could play a role: the longer time required to eliminate such cells with an antimicrobial treatment could give the cells enough time to go through these different steps and gain full resistance. Biofilm tends to promote physiological heterogeneity and cell tolerance due to its 3D structure, which can work in favour of the proportion of tolerant cells in the population, thereby substantially contributing to the emergence of resistance against biocides.

Simultaneously, high mutation rates have been associated with biofilms [132], specifically in persister populations [133], hence increasing the chances of acquiring genetic resistances. This could be due to the use of error-prone polymerases during the SOS response, which ultimately leads to higher probability of mutational events and fixation of some resistance mechanisms [134, 135]. Deletion of the SOS response activator RecA has already been associated with a delay in the acquisition of antibiotic resistance in *E. coli* [136], and could therefore play a major role in the acquisition of biocide resistance.

Physiological responses to biocide exposure can also stimulate mutation rates. The addition of chlorhexidine, didecyldimethylammonium chloride (DDAC), copper, permethrin and propiconazole have been demonstrated to activate global stress responses, such as the RpoS-mediated response and the SOS response in *E. coli *, subsequently increasing the mutation rate [137]. Oxidizing agents such as H_2_O_2_ and PAA can also induce the formation of ROS [138, 139] that are toxic for the cells and damage DNA. Nevertheless, the protection conferred by the biofilm could limit exposure effects so they remain sublethal. These concentrations could increase DNA instability, which would ultimately lead to mutations, but the concentrations would not be high enough to be lethal for the bacterial cell. Exposure to H_2_O_2_ has, for example, been shown to markedly increase the mutation rates in yeasts [140].

Finally, an increase in mutation rate has also been associated with efflux pumps. A recent study associated the upregulation of *acrB* with a higher mutation rate [141]. This efflux pump is one of the most strongly associated with antimicrobial survival and, as previously noted, has already been linked to biocide tolerance [108]. Hence, its overexpression would allow the bacteria to both survive biocide exposure and simultaneously induce mutations in the population.

### Triggering of genetic exchanges

Biofilms are hotspots for horizontal gene transfers (HGTs) between bacteria [142]. The density of bacteria is higher than in planktonic cultures and bacteria stay physically close together, thus increasing the probability of DNA exchange. This can happen in mono-species biofilms but also between two different species in multi-species biofilms, which are frequent in natural environments [143].

Different transfer mechanisms support HGTs, and bacterial conjugation is one of the best described. Proximity between bacteria facilitates intercommunication. It has already been shown that plasmids are better conserved in biofilms [144] and that exchanges are also fostered by other mechanisms intrinsic to biofilms, such as the SOS response, which is interlinked with bacterial conjugation [145, 146]. In addition, the 3D structure of biofilms may facilitate plasmid stability and persistence even under non-selective conditions [147, 148].

Reciprocally, plasmids can also contribute to biofilm formation and development. Conjugative pili are, for example, known to facilitate biofilm formation thanks to their aggregative properties, which improve cell–cell fixation properties [147]. Ghigo [149] demonstrated that conjugative pili could also increase cell-surface adherence and increase the chances of initial attachment. This could be due to the upregulation of curli and colanic acid production associated with the conjugative plasmid [150]. While conjugative pili are naturally present on every plasmid as a backbone gene essential for the plasmid’s horizontal transmission, other genes able to facilitate biofilm formation have been found on some plasmids. Of these, fimbriae and type IV pili — important genes in the adhesion step — are frequently found on plasmids [147].

Resistance plasmids are of concern because of their fast and efficient exchange of resistance genes. Additionally, the use of a single antimicrobial substance can select for multidrug-resistant bacteria as plasmids often carry a large number of genes, conferring broad resistances. Some biocide resistance genes have already been observed on plasmids [6, 151–155] and could spread easily between different bacterial populations. Moreover, the addition of certain biocides, such as chlorine, chloramine and hydrogen peroxide, could directly enhance conjugation events, probably by inducing bacterial stress responses [156].

While the acquisition of bacterial plasmids could thus be facilitated in biofilms, other HGT methods can also be induced. Bacteria may also acquire resistance genes through the transformation of genetic material. The large quantities of eDNA in the biofilm matrix can be acquired by neighbouring bacteria and hence spread acquired bacterial resistance mechanisms. Indeed, bacterial transformation is generally induced inside biofilms due to the eDNA in their matrix [157].

Furthermore, by lysing their targets, biocides could actually induce this type of genetic exchange. Indeed, many biocides lyse cells by inducing a loss of membrane integrity, thereby releasing intracellular content into the biofilm matrix [158, 159]. Some tolerant bacteria that survived exposure would be able to transform some of the genetic material released, and could thereby acquire resistance genes [160–162].

### Protective incubation of newborn variants

The superposition of metabolic and biocide gradients resulting from the 3D structure increase the chances of tolerant subpopulations developing resistance without being eliminated by the treatment. However, resistant phenotypes are often associated with a fitness cost that can be caused by a mutation of the bacterial target, inhibiting the effect of the biocide, but also altering its function. It can also be a consequence of elevated energy costs, brought about by efflux pump upregulations [163] or the addition of a plasmid to the bacterial genome [164]. New research, however, tends to characterize biofilms as ‘diversity incubators’ [165], boosting the appearance of variants and greatly helping them to survive and settle in the population. In addition to their ability to promote the appearance of variants, biofilms could play a major role in the fixation of these variants in the environment. Indeed, protective microenvironments will permit variants to survive in biofilms, while in a well-mixed suspension they would probably be directly outcompeted by strains with higher fitness once selective pressure is removed after the end of biocide exposure. The cooperative behaviour in biofilms and the protective effects of the structure will actually provide mutants with the opportunity to survive and develop, potentially allowing the acquisition of additional mutations in daughter cells. Consequently, some of these bacteria will earn compensatory mutations, which will restore or even increase their fitness, hence allowing mutants to fix in the population, and be able to disseminate and colonize new environments. France *et al*. have studied the fixation of gentamicin- and rifampicin-resistant mutants in biofilm and planktonic populations after initial antibiotic exposure [166]. They showed that the gentamicin-resistant clones, which suffered severe fitness costs, could remain in the same quantities in biofilms even 45 days after the antibiotic exposure, while the number of resistant variants in planktonic conditions declined greatly over time. On the other hand, the rifampicin-resistant clones, whose mutations do not usually involve fitness costs, could persist in the same quantities in both planktonic and biofilm conditions.

## Impact of bacterial adaptation to biocides in the light of the One Health concept

Recent health crises have increased the general public’s concern about food processing procedures. The impacts of such procedures, in particular hygiene and disinfection, need to be investigated from farm to fork in the light of the One Health concept, which recognizes that human, animal and environmental health are intricately linked. Hence, studies on the side effects of bacterial adaptation to biocides have to be enforced to identify potential impacts on and risks to public health issues. More especially, the biofilm model needs to be integrated because it is a main way that bacteria can adapt along the food chain [167]. This section will thus summarize the main possible effects of biocide use on pathogens that can have a food safety impact from a One Health perspective.

### Colonization and dissemination

Biocide use may affect the ability of the exposed bacterial populations to disseminate and colonize new environments, especially after several industrial disinfection cycles. Any misuse of biocides could increase the dissemination of viable cells by the superficial disruption of the biofilm without being strong enough to eradicate bacterial cells inside the biofilm. The effects of BAC and NaOCl on *L. monocytogenes* were studied by Rodriguez-Melcon *et al*. [168], who found that the use of both biocides at 0.5, 1.0 and 1.5 MICs could reduce the biofilm’s biovolumes of the different strains. However, only NaOCl was able to kill the vast majority of the cells, while MICs of BAC were not able to reduce the number of viable cells. The same effects have been observed with PAA and BAC on *Yersinia enterocolitica* and *Cronobacter sakazakii* [169]. A sub-optimal biocide treatment could thereby lead to a detachment of viable cells that could easily recolonize new surfaces.

An increase in adhesion properties has also been observed in sulphate-reducing bacteria after exposure to glutaraldehyde [170]. Likewise, NaOCl and acetic acid could enhance the surface adhesion force of *Campylobacter jejunii* [171]. This can be explained by the overexpression of adhesion-related genes [172, 173] and the downregulation of motility-related genes [174]. New surface colonization could thereby be facilitated for the biofilm-detached exposed cells.

Finally, several studies have already associated biocide exposure with changes in biofilm formation, thus potentially increasing the faculty of bacteria to form stronger biofilms on the newly colonized surfaces. A study on uropathogenic *E. coli* has shown a positive association between biofilm formation and both triclosan and BAC [33]. A positive association with biofilm formation has also been reported for sodium nitrite and NaOCl in *E. coli* [34], while *Salmonella enteritidis* biofilm capabilities have been enhanced upon exposure to NaOCl and H_2_O_2_ at sublethal concentrations [175].

Food processing industries usually employ high concentrations of biocides during cleaning procedures, which should prevent these events from happening. However, the presence of biofilms, combined with other limitations related to surface topology, the presence of interfering matter, or even misuse, for instance, could lead to a decrease in the final biocide concentrations applied to bacteria. Adaptation to such low biocide concentrations may thus provide bacteria with an opportunity to propagate in the food chain through a higher adhesion or biofilm production capabilities, thus increasing the chance of reaching the consumer at the end of the process.

### Antimicrobial cross-resistance

The possible dissemination of biocide-exposed pathogens along the food chain towards consumers raises concerns about the impact of biocide exposure on antibiotic resistance. This potential side effect of biocides has been gaining interest due to the growing threat of antimicrobial resistance worldwide. Numerous classes of biocides and antibiotics have indeed been associated with cross-resistances [9], notably because of the similar mechanisms bacteria use to fight against both kinds of molecules (see Table 1). Multidrug efflux pump overexpression is one of the main co-selection mechanisms reported between biocides and antibiotics. As previously stated, efflux pumps play an important role in biofilm formation and defence against biocides, but their non-specificity also leads them to play an important role in antibiotic resistance. Several studies have directly associated some efflux pumps with cross-resistance against different classes of biocides and antibiotics, in various foodborne pathogens [176, 177]. Cross-resistance through modifications to cell permeability via porin mutations has also been documented [176, 178, 179]. Changes in metabolism can also affect both tolerance to biocides and antibiotics, which is particularly relevant in biofilms, as shown in 2021 by Cuzin *et al*., who exposed *E. coli* biofilms to PHMB and isolated a gentamicin-resistant variant with lower susceptibility to PHMB from the exposed biofilm population [15]. Decreases in antimicrobial susceptibility are related to a single nonsense mutation in the gene *aceE* encoding the pyruvate dehydrogenase E1 component of the pyruvate dehydrogenase (PDH) complex that catalyses the conversion of pyruvate to acetyl-CoA and CO_2_ upstream of the tricarboxylic acid cycle [15].

**Table 1.** List of genetic targets directly or indirectly associated with a decreased antibiotic susceptibility due to biocide cross-selection

Modulation of cellular functions underlying cross-resistance	Genetic target	Biocidal active substances	Reduced antibiotic susceptibility	Bacterial species	Reference
Efflux activity	AbeM	TCS	Cip, Dox, Min	* Acinetobacter baumannii *	[129]
	AcrAB/TolC	FOR+GLU+QAC	Acr, Amp, Cm, Tet	* Salmonella enterica *	[197]
		TCS	Acr, Amp, Cm, Tet	* Salmonella enterica *	[197]
		FOR+GLU+QAC	Amp, Cip, Cm, Tet	* Salmonella enterica *	[200]
		FOR+GLU+QAC	Amp, Cip, Cm, Tet	* Salmonella enterica *	[200]
		OCB	Amp, Cip, Cm, Tet	* Salmonella enterica *	[200]
		OCB	Amp, Cip, Cm, Tet	* Salmonella enterica *	[200]
	AcrEF/TolC	ALD+QAC	Cm, Cip, Nal, Tet	* Salmonella enterica *	[201]
		OCB	Cm, Cip, Nal, Tet	* Salmonella enterica *	[201]
	AcrR	BAC	Amp, Cm	* Escherichia coli *	[7]
		TCS	Amp, Cm, Lev	* Escherichia coli *	[202]
		TCS	Amp, Azi, Cfc, Cfp, Cfz, Cxm, Cip, Ery, Gen, Lin, Lom, Oxa, Tet	* Escherichia coli *	[12]
		BAC	Amp, Cm, Tet	* Escherichia coli *	[203]
		DDAC	Amp, Cm, Tet	* Escherichia coli *	[203]
	AdeB	CHX	Ami, Cip, Dox, Gen, Imi, Mer, Min, Net, Sul, Tob	* Acinetobacter baumannii *	[129]
		BAC	Ami, Cip, Dox, Gen, Imi, Mer, Min, Net, Sul, Tob	* Acinetobacter baumannii *	[129]
	AdeJ	TCS	Cip, Dox, Min	* Acinetobacter baumannii *	[129]
	AdeS	CHX	Ami, Cip, Dox, Gen, Imi, Mer, Min, Net, Sul, Tob	* Acinetobacter baumannii *	[129]
		BAC	Ami, Cip, Dox, Gen, Imi, Mer, Min, Net, Sul, Tob	* Acinetobacter baumannii *	[129]
	CepA	CHX	Col	* Klebsiella pneumoniae *	[204]
	CmeB	TCS	Amp, Cip, Cm, Ery, Tet,	* Campylobacter jejuni *	[205]
	DinF	DDAC	Amp, Tet	* Escherichia coli *	[203]
	FepR	BAC	Cip	* Listeria monocytogenes *	[10]
	MdfA	CHP	Amp, Cm	* Escherichia coli *	[7]
		TCS	Amp, Azi, Cfc, Cfp, Cfz, Cxm, Cip, Ery, Gen, Lin, Lom, Oxa, Tet	* Escherichia coli *	[12]
	MepA	BAC	Acr, Cip, Nor	* Staphylococcus aureus *	[206]
		CHX	Acr, Cip, Nor	* Staphylococcus aureus *	[206]
		CHX	Cip, Mup	* Staphylococcus aureus *	[116]
	MexAB/OprN	TCS	Cip, Ery, Gen, Tet, Tmp	* Pseudomonas aeruginosa *	[207]
	MexCD/OprJ	BAC	Nor	* Pseudomonas aeruginosa *	[208]
		CHX	Nor	* Pseudomonas aeruginosa *	[208]
		TCS	Cip, Ery, Tet, Tmp	* Pseudomonas aeruginosa *	[207]
	MexX	CHX	Ami, Cef, Cip, Mer	* Pseudomonas aeruginosa *	[209]
	MexXY/OprM	TCS	Cip, Ery, Gen, Tet, Tmp	* Pseudomonas aeruginosa *	[207]
	MsbA	DDAC	Amp, Cm, Tet	* Escherichia coli *	[203]
	NorA	CHX	Cip, Mup	* Staphylococcus aureus *	[116]
	NorE	TCS	Amp, Azi, Cfc, Cfp, Cfz, Cxm, Cip, Ery, Gen, Lin, Lom, Oxa, Tet	* Escherichia coli *	[12]
	OqxAB	BAC	Cip, Cm, Flu, Nal, Nor, Tmp	* Escherichia coli *	[210]
		TCS	Cip, Cm, Flu, Nal, Nor, Tmp	* Escherichia coli *	[210]
	PmpM	BAC	Acr, Cip, Nor	* Pseudomonas aeruginosa *	[211]
	SmeDEF	TCS	Cip, Cm, Tet	* Stenotrophomonas maltophilia *	[212]
	YihV	TCS	Amp, Azi, Cfc, Cfp, Cfz, Cxm, Cip, Ery, Gen, Lin, Lom, Oxa, Tet	* Escherichia coli *	[12]
Membrane permeability	CarO	BAC	Ami, Cip, Dox, Gen, Imi, Mer, Min, Net, Sul, Tob	* Acinetobacter baumannii *	[129]
		TCS	Ami, Cip, Dox, Gen, Min, Net	* Acinetobacter baumannii *	[129]
	EnvZ	CHP	Amp, Cm, Nor	* Escherichia coli *	[7]
	FabH	TCS	Cip	* Staphylococcus aureus *	[213]
	MipA	BAC	Amp, Cm, Tet	* Escherichia coli *	[203]
	OmpA	OCB	Amp, Cip, Cm, Tet	* Salmonella enterica *	[200]
		FOR+GLU+QAC	Amp, Cip, Cm, Tet	* Salmonella enterica *	[200]
		BAC	Ami, Cip, Dox, Gen, Imi, Mer, Min, Net, Sul, Tob	* Acinetobacter baumannii *	[129]
		CHX	Ami, Cip, Dox, Gen, Imi, Mer, Min, Net, Sul, Tob	* Acinetobacter baumannii *	[129]
		TCS	Ami, Cip, Dox, Gen, Min, Net	* Acinetobacter baumannii *	[129]
	OmpC	OCB	Amp, Cip, Cm, Tet	* Salmonella enterica *	[200]
		FOR+GLU+QAC	Amp, Cip, Cm, Tet	* Salmonella enterica *	[200]
	OmpF	OCB	Amp, Cip, Cm, Tet	* Salmonella enterica *	[200]
		FOR+GLU+QAC	Amp, Cip, Cm, Tet	* Salmonella enterica *	[200]
	OmpR	CHP	Amp, Cm, Nor	* Escherichia coli *	[7]
		POV	Amp, Cm, Nor	* Escherichia coli *	[7]
	PmrB	BAC	Pol	* Pseudomonas aeruginosa *	[14]
	SbmA	BAC	Amp, Cm, Tet	* Escherichia coli *	[203]
Central metabolism	AceE	PHMB	Gen	* Escherichia coli *	[15]
Biofilm formation	Aes	GLU	Cm	* Escherichia coli *	[7]
	Flu	CHX	Amp	* Escherichia coli *	[7]
	PyrE	GLU	Amp, Cm, Nor	* Escherichia coli *	[7]
	YeaW	GLU	Amp, Cm, Nor	* Escherichia coli *	[7]
Modification of specific target	FabI	TCS	Cip	* Staphylococcus aureus *	[213]
		HAL	Cip, Nal	* Salmonella enterica *	[8]
		OCB	Cip, Nal	* Salmonella enterica *	[8]
	GrlA	TCS	Cip	* Staphylococcus aureus *	[213]
	GrlB	TCS	Cip	* Staphylococcus aureus *	[213]
	GyrA	TCS	Cip	* Staphylococcus aureus *	[213]
		HAL	Cip, Nal	* Salmonella enterica *	[8]
		OCB	Cip, Nal	* Salmonella enterica *	[8]
	InhA	TCS	Iso	* Mycobacterium tuberculosis *	[214]
	RpoA	AQAS	Cm, Nal, Tet	* Salmonella enterica *	[8]
		GLU	Cm	* Escherichia coli *	[7]
	RpoB	PER	Amp, Cm	* Escherichia coli *	[7]
		BAC	Amp	* Escherichia coli *	[7]
		BAC	Rif	* Escherichia coli *	[203]
		DDAC	Rif	* Escherichia coli *	[203]
	RpoC	PER	Amp, Cm	* Escherichia coli *	[7]
		BAC	Rif	* Escherichia coli *	[203]
		DDAC	Rif	* Escherichia coli *	[203]
Global regulator activity	Crp	BAC	Amp, Cm, Tet	* Escherichia coli *	[203]
		DDAC	Amp, Cm, Tet	* Escherichia coli *	[203]
	GdpP	SHY	Oxa	* Staphylococcus aureus *	[215]
	Lon	BAC	Amp, Cm, Tet	* Escherichia coli *	[203]
	FrdD	TCS	Amp, Amx, Cep	* Escherichia coli *	[202]
	MarA	BAC	Cip, Cm, Ctf, Cxm, Flo, Nal	* Escherichia coli *	[128]
	MarR	TCS	Cm, Lev, Nor, Tet	* Escherichia coli *	[202]
		BAC	Amp, Cm, Tet	* Escherichia coli *	[203]
		DDAC	Amp, Cm, Tet	* Escherichia coli *	[203]
	PhoPQ	CHX	Col	* Klebsiella pneumoniae *	[198]
	RamR	QAC	Cm, Nal, Tet	* Salmonella enterica *	[8]
		ALD+QAC	Cm, Nal, Tet	* Salmonella enterica *	[8]
	SoxR	TCS	Amp, Cm, Lev	* Escherichia coli *	[202]
		BAC	Amp, Cm, Tet	* Escherichia coli *	[203]
		DDAC	Amp, Cm, Tet	* Escherichia coli *	[203]
	SoxS	BAC	Cip, Cm, Ctf, Cxm, Flo, Nal	* Escherichia coli *	[128]

Acr, acriflavine; ALD, aldehydes; Ami, amikacin; Amp, ampicillin; Amx, amoxicillin; Azi, azithromycin; BAC, benzalkonium chloride; Cep, cephalexin; Cfc, cefaclor; Cfp, cefepime; Cfz, Cefazolin; CHP, chlorophene; CHX, chlorhexidine; Cip, ciprofloxacin; Cm, chloramphenicol; Col, colistin; Ctd, ceftiofur; Cxm, cefotaxime; DDAC, didecyldimethylammonium chloride; Dox, doxycycline; Ery, erythromicin; Flu, flumequine; FOR, formaldehyde; Gen, gentamicin; GLU, glutaraldehyde; HAL, chlorophene tertiary amine compound; Imi, imipenem; Iso, isoniazid; Lev, levofloxacin; Lin, linezolid; Lom, lomefloxacin; Mer, meropenem; Min, minocycline; Mup, mupirocin; Nal, nalidixic acid; Net, netilmicin; Nor, norfloxacin; OCB, oxidising compound blend; Oxa, oxacillin; PER, hydrogen peroxide; PHMB, polyhexamethylene biguanide; PIO, piovidone iodine; Pol, polymixin B; QAC, quaternary ammonium compound; Rif, rifampicin; Sul, sulbactam; TCS, triclosan; Tet, tetracycline; Tmp, trimethoprim; Tob, tobramycin.

Additionally, plasmids containing genes conferring resistance to both biocides and antibiotics have already been identified, notably QAC efflux pumps that are often found on multidrug resistance plasmids [151, 155, 180–182]. However, numerous studies have also failed to identify a direct link between biocide exposure and an increase in antibiotic resistance [5, 183, 184]. Another study by Li *et al*. showed that, under their conditions, resistance to triclosan could induce some physiological modifications which conferred tolerance to antibiotics by inducing the formation of biofilm and reducing membrane permeability, without, however, inducing irreversible modifications which would lead to permanent resilience [185]. The link between both antimicrobial classes is not always clear and the emergence of resistance to antibiotics has already been observed after biocide exposure without having any effect on the biocide MICs [186, 187]. Finally, biocide exposure can even increase the susceptibility of bacteria to some antibiotic classes [183, 188], with some resistance mutations having occasional global effects on the bacterial cell that disrupt the effect of other mechanisms [189]. Hence, additional studies are needed to improve our understanding of the role of biocides in the emergence of antibiotic resistance from a One Health perspective.

### Virulence and pathogenicity

Another interesting line of research in keeping with a One Health approach is the study of the potential impact of biocides on the virulence and pathogenicity of strains. The global risk of consumer contamination would be instantly increased if adaptation to biocides likewise increases the strain’s ability to infect its host. One of the impacts that biocides have on efflux pumps could, for example, be increased virulence. Several efflux systems have been associated with the ability of some pathogens to infect and colonize human cells [190]. The AcrAB-TolC complex has been directly linked to the faculty of *S. enterica* serovar Typhimurium to infect macrophage cells [191], probably by controlling the expression of pathogenicity islands SPI-1 and SPI-2 [192]. It has also been associated with survival against bile salts found in the gastrointestinal tract [193]. The invasiveness of *P. aeruginosa* has been shown to be greatly reduced following mutation of the MexAB-OprM system [194]. ROS-degrading enzymes are also important proteins in both biocide resistance and virulence. The RcsA peroxidase of *P. aeruginosa* has been found to be associated with both *in vivo* bacterial survival in *Caenorhabditis elegans* and *Drosophila melanogaster* and an increase in the survival rate against sodium hypochlorite and hypochlorous acid [195]. However, despite the different possible links between virulence and biocide resistance, the studies that have investigated virulence after exposure to biocides did not find any positive impact of biocide exposure on the pathogens’ natural virulence. Exposure of *S. enterica* serovar Typhimurium to several classes of biocides (a tar oil phenol, an oxidizing agent, an aldehyde agent and a QAC agent) has been found not to affect virulence in chicks [196]. Another study performed on *S. enterica* serovar Typhimurium has even shown a significantly reduced invasiveness of triclosan-exposed and QAC-exposed bacterial cells [197]. Henly *et al*. [33] showed more mixed results on uropathogenic *E. coli *. Triclosan exposure was indeed found to reduce virulence in 5/8 strains and BAC in 6/8 strains although virulence of the least pathogenic strain was nevertheless induced after BAC exposure. Contrasting results were also observed with PHMB (1/8 showed increased virulence and 3/8 reduced virulence). The last biocide tested, silver nitrate, was actually the only one that never reduced pathogenicity, and in fact increased it in 2/8 strains tested. Wand *et al*. [198] also showed contrasting results with 4/6 strains that had a significant loss of virulence after CHX exposure while the last two did not seem to have an impact on the survival of *Galleria mellonella* larvae. Another study has shown that hypervirulent strains of *L. monocytogenes* were positively associated with a plasmid carrying the *emrC* gene coding BAC efflux pumps, which could show a positive selection of virulent clones through the use of this biocide, but no direct link has been made to confirm the efflux pump’s direct role on the virulence of these strains [199]. To conclude, no general link between biocide exposure and virulence has yet been proven, but the fitness costs induced by triclosan and QAC exposure seem to have, in general, a deleterious effect on cells. More studies will be required on different biocide classes to identify potential links between pathogenicity and biocide adaptation.

## Conclusion

Considerable progress has been made in the last few decades on our understanding of how biofilms adapt to their environment. The heterogeneity of phenotypes caused by the 3D structure facilitates tolerance against external stresses. New studies are exploring the link between bacterial tolerance and the appearance of acquired resistances, paving the way for future research on a possible link between biofilms and the propagation of harmful bacterial variants. The constant exposure of biofilms to biocides in food chain industries raises the question of potential effects of biocide exposure on the development and propagation of antimicrobial resistance variants via the food chain, resulting in a possible public health risk. Field studies linking all these characteristics in natural environments are required to evaluate the risk associated with these phenomena.
